# Projected Life Expectancy Gains From Improvements in HIV Care in Black and White Men Who Have Sex With Men

**DOI:** 10.1001/jamanetworkopen.2023.44385

**Published:** 2023-11-28

**Authors:** Katherine M. Rich, Ankur Pandya, John J. Chiosi, Krishna P. Reddy, Fatma M. Shebl, Andrea L. Ciaranello, Anne M. Neilan, Jodian A. Pinkney, Elena Losina, Kenneth A. Freedberg, Aima A. Ahonkhai, Emily P. Hyle

**Affiliations:** 1 Medical Practice Evaluation Center (MPEC), Massachusetts General Hospital, Boston; 2 Harvard Medical School, Boston, Massachusetts; 3 Department of Health Policy and Management, Harvard T.H. Chan School of Public Health, Boston, Massachusetts; 4 Division of Infectious Diseases, Department of Medicine, Massachusetts General Hospital, Boston; 5 Tobacco Research and Treatment Center, Massachusetts General Hospital, Boston; 6 Division of Pulmonary and Critical Care Medicine, Massachusetts General Hospital, Boston; 7 Division of General Academic Pediatrics, Massachusetts General Hospital, Boston; 8 Department of Biostatistics, Boston University School of Public Health, Massachusetts; 9 Orthopedic and Arthritis Center for Outcomes Research (OrACORe), Department of Orthopedic Surgery, Brigham and Women’s Hospital, Boston, Massachusetts; 10 Policy and Innovation Evaluation in Orthopedic Treatments (PIVOT) Center, Department of Orthopedic Surgery, Brigham and Women’s Hospital, Boston, Massachusetts; 11 Division of General Internal Medicine, Department of Medicine, Massachusetts General Hospital, Boston; 12 Harvard University Center for AIDS Research, Harvard University, Boston, Massachusetts; 13 Department of Medicine, Infectious Diseases, Vanderbilt University Medical Center, Nashville, Tennessee; 14 Vanderbilt Institute for Global Health, Vanderbilt University Medical Center, Nashville, Tennessee

## Abstract

**Importance:**

Substantial racial inequities exist across the HIV care continuum between non-Hispanic Black and White men who have sex with men (MSM) in the US.

**Objectives:**

To project years of life gained (YLG) with improving the HIV care continuum among Black MSM and White MSM in the US and to determine the outcomes of achieving health equity goals.

**Design, Setting, and Participants:**

The Cost-Effectiveness of Preventing AIDS Complications microsimulation model was used and populated with 2021 race-specific data to simulate HIV care among Black MSM and White MSM in the US who have acquired HIV. Analyses were completed from July 2021 to October 2023.

**Intervention:**

The study simulated status quo care using race-specific estimates: age at infection, time to diagnosis, receipt of care, and virologic suppression. The study next projected the outcomes of attaining equity-centered vs non–equity-centered goals by simulating 2 equal improvements in care goals: (10-point increased receipt of care and 5-point increased virologic suppression), 3 equity-centered goals (annual HIV testing, 95% receiving HIV care, and 95% virologic suppression) and lastly, an equitable care continuum that achieves annual HIV testing, 95% receiving care, and 95% virologic suppression in Black MSM and White MSM. One-way and multiway sensitivity and scenario analyses were conducted.

**Main Outcomes and Measures:**

Mean age at death and YLG.

**Results:**

In the simulated cohort, the mean (SD) age at HIV infection was 27.0 (10.8) years for Black MSM and 35.5 (13.6) years for White MSM. In status quo, mean age at death would be 68.8 years for Black MSM and 75.6 years for White MSM. The equal improvements in care goals would result in 0.5 YLG for Black MSM and 0.5 to 0.9 YLG for White MSM. Achieving any 1 equity-centered goal would result in 0.5 to 1.7 YLG for Black MSM and 0.4 to 1.3 YLG for White MSM. With an equitable care continuum compared with the nationally reported status quo*,* Black MSM and White MSM would gain 3.5 and 2.1 life-years, respectively. If the status quo HIV testing was every 6 years with 75% retained in care and 75% virologically suppressed, Black MSM would gain 4.2 life-years with an equitable care continuum*.*

**Conclusions and Relevance:**

In this simulation modeling study of HIV care goals, equal improvements in HIV care for Black and White MSM maintained or worsened inequities. These results suggest that equity-centered goals for the HIV care continuum are critical to mitigate long-standing inequities in HIV outcomes.

## Key Points

**Question:**

Given existing inequities in the HIV care continuum, what are the differences in age at death between Black and White men who have sex with men (MSM) who acquire HIV, and what would be the outcome of achieving equity-centered goals?

**Findings:**

Using a decision analytical model, the mean age at death would be 68.8 years for Black MSM and 75.6 years for White MSM given the current HIV care continuum. If HIV care is improved by equal increments for Black and White MSM, racial inequities would be the same or worsened, whereas achieving equity-centered HIV care goals would be a step toward achieving health parity between Black and White MSM.

**Meaning:**

These findings suggest investment in equity-centered HIV care goals is critical to mitigate long-standing inequities in HIV care outcomes.

## Teaser

This decision analytical model evaluates the projected life expectancy gains from improvements in HIV care in men who have sex with men.

## Introduction

Substantial inequities persist across the HIV care continuum in the US, with Black people with HIV (PWH) bearing a disproportionate disease burden due, in part, to structural factors such as stigma, racism, and other social determinants of health.^1,2,3,4,5,6^ Black PWH are less likely to receive prompt diagnosis, consistently receive HIV care, and achieve virologic suppression than White PWH.^2^ These inequities are particularly evident between Black men who have sex with men (MSM) and White MSM. Less than 85% of Black MSM with HIV receiving care were virologically suppressed in 2021 compared with more than 90% of White MSM with HIV; virologic nonsuppression increases morbidity, mortality, and HIV transmission.^1^ Reasons for these inequities are multifactorial and rooted in social and structural factors, including but not limited to legacies of slavery, mass incarceration, income inequality, and community-level stigma.^7,8,9^ Inequities in access to health care have continued to persist in large part due to people being uninsured and underinsured, which disproportionately affects Black communities.^10^

Reducing inequities in the HIV care continuum is 1 of the 4 key tenets of the National HIV/AIDS Strategy.^11,12,13^ This initiative aligns with growing calls for local and national programs to set HIV care goals that promote health equity, or the attainment of the highest level of health for all people.^14,15^ In contrast, goals that aim for similar increments in improvement in care across subpopulations, rather than setting identical outcome goals, allow racial inequities to persist.

To achieve health equity, investments must be made in interventions that address multilevel factors driving racial inequities, as well as evaluations of public health goals to determine effective strategies. We used simulation modeling to assess the potential implications of different HIV care continuum goals by examining which strategies would reduce gaps in life expectancy and improve outcomes among Black MSM and White MSM with HIV. Although prevention efforts, such as preexposure prophylaxis (PrEP), are essential to comprehensive care, we explicitly focused this study on improving care among MSM living with HIV to guide efforts toward strengthening the care continuum for individuals with HIV.

## Methods

### Analytic Overview

The Cost-Effectiveness of Preventing AIDS Complications (CEPAC) microsimulation model was used to project survival for non-Hispanic Black MSM with HIV and non-Hispanic White MSM with HIV given 7 different HIV care continuum goals. First, we populated the status quo care strategy with race-specific national estimates of testing frequency, the proportion of MSM with diagnosed HIV receiving care, and the proportion with virological suppression. We then simulated 2 equal improvements in care goals, which would attain equal absolute improvements in care between Black MSM and White MSM: (1) 10-point increased receipt of care and (2) 5-point increased virologic suppression (eFigure 1 in Supplement 1). We next simulated 3 equity-centered goals, in which equity between Black MSM and White MSM is achieved in 1 step of the HIV care continuum: (1) annual HIV testing for earlier diagnosis, (2) 95% receiving HIV care among MSM with diagnosed HIV, and (3) 95% virologic suppression among MSM receiving HIV care (eFigure 2 in Supplement 1). Last, we simulated an equitable care continuum that achieves annual HIV testing, 95% receiving HIV care, and 95% virologic suppression.^16^ These goals align with recommendations from the National HIV/AIDS Strategy and the US Centers for Disease Control and Prevention (CDC).^11,17,18^ To examine the association between racial inequities within the HIV care continuum and HIV care outcomes, we projected the mean age at death and compared the mean years of life gained (YLG) from attaining each HIV care continuum goal for Black MSM and White MSM with HIV.

### The CEPAC Model

The CEPAC model is a validated Monte Carlo microsimulation model of HIV disease and treatment.^16,19,20,21^ Simulated individuals experience clinical events, including HIV infection, diagnosis, and virologic suppression (eAppendix and eFigure 3 in Supplement 1). Research projects using the CEPAC model are approved by the Mass General Brigham human research committee; we used the Consolidated Health Economic Evaluation Reporting Standards (CHEERS) reporting guidelines. The need for informed consent was waived in this study as no individual-level data were used. All data used in this study to create model parameters were either aggregate data from the US Centers for Disease Control and Prevention or from peer-reviewed literature.

#### HIV Acquisition, HIV Diagnosis, and ART Initiation

Following infection with HIV, individuals experience a monthly decline in CD4 count while not receiving antiretroviral therapy (ART), as well as CD4-stratified risks of opportunistic infections and HIV-related mortality. HIV diagnosis is determined by a race-specific monthly probability of testing. After diagnosis, an individual can initiate ART with an integrase strand transfer inhibitor–based regimen.^22^ While on ART, individuals have a rise in CD4 count and a decreased risk of opportunistic infections and other HIV-related mortality.

#### ART Adherence and Outcomes for HIV Care

 Higher ART adherence results in a lower risk of disengaging from care and a greater probability of virologic suppression.^23^ Individuals disengaged from care have a decline in CD4 count; they also have a monthly probability of returning to HIV care.^24^

#### Mortality

HIV-related mortality depends on an individual’s CD4 count. Individuals are assigned age-specific and race-specific non–HIV-related mortality rates derived from national life tables, adjusted for smoking prevalence.^25^

### Model Input Parameters

We populated the status quo strategy with race-specific estimates of the 2021 HIV care continuum using national data from the CDC HIV Surveillance Reports (Table).^1,26^ We estimated the mean time from HIV infection until diagnosis among Black MSM and White MSM, given reported CD4 counts at diagnosis (eTable 1 in Supplement 1).^1,26,27^ We subtracted the mean diagnostic delay from the mean age at HIV diagnosis to estimate the mean (SD) age at HIV infection: 27.0 (10.8) years for Black MSM and 35.5 (13.6) years for White MSM (Table). We calibrated the frequency of HIV testing among Black MSM (every 4.9 years) and White MSM (every 4.6 years) to the estimated time until HIV diagnosis.^27^

**Table.** Model Input Parameters and Calibration Targets for Simulated Populations of Black and White Men Who Have Sex With Men (MSM) With HIV

Parameter	Non-Hispanic Black MSM	Non-Hispanic White MSM	Source
HIV care continuum with status quo care			
Age at HIV infection, mean (SD), y	27.0 (10.8)	35.5 (13.6)	Derived from CDC,^1^ 2021; CDC,^26^ 2023; CDC,^27^ 2017.
Time from HIV infection until diagnosis, mean (95% CI), y	3.4 (3.3-3.6)	3.3 (3.1-3.5)	Derived from CDC,^1^ 2021; CDC,^26^ 2023; CDC,^27^ 2017.
Age at HIV diagnosis, mean (SD), y	30.4 (10.8)	38.8 (13.6)	CDC,^26^ 2023.
Mean HIV testing frequency, y	Every 4.9	Every 4.6	Derived from CDC,^1^ 2021; CDC,^26^ 2023; CDC,^27^ 2017.
CD4 at ART initiation, mean (SD), cells/μL	380 (195)	373 (209)	CDC^1^ 2021.
Receipt of care after diagnosis, %	75.3	80.3	CDC^1^ 2021.
Virologic suppression among MSM receiving care, %	83.6	92.4	CDC^1^ 2021.
Mean time lost to follow-up, y	1.9	2.6	Derived from Wang et al,^24^ 2019.
Ranges of annualized non–HIV-related mortality (deaths/y), stratified by age group^a^			
<30 y	≤0.0026	≤0.0018	Derived from Reddy et al,^25^ 2016; Arias et al,^29^ 2019; and Mdodo et al,^30^ 2015.
30-39 y	0.0027-0.0041	0.0019-0.0026	
40-49 y	0.0027-0.0083	0.0017-0.0054	
50-59 y	0.0089-0.0188	0.0058-0.0123	
≥60 y	≥0.0203	≥0.0133	

Abbreviations: ART, antiretroviral therapy; CDC, US Centers for Disease Control and Prevention.

^a^ Life tables adjusted for tobacco smoking prevalence.

We calibrated the model to achieve race-specific estimates of receipt of care and virologic suppression: 75.3% (Black MSM) and 80.3% (White MSM) with diagnosed HIV received HIV care in 2021, and 83.6% (Black MSM) and 92.4% (White MSM) receiving care were virologically suppressed (eTable 2 and eTable 3 in Supplement 1).^1,26,28^ We defined receipt of care as documentation of 1 or more CD4 or viral load tests during 2021 and virologic suppression as a viral load level less than 200 copies/mL.^1^

We derived the age-stratified monthly non–HIV-related mortality rates from non-Hispanic Black and White male US national life tables.^29^ Given the higher prevalence of tobacco smoking among PWH compared with the general adult population, we adjusted these estimates for excess mortality attributable to tobacco smoking among MSM with HIV according to race-specific tobacco smoking prevalence (Table; eTable 4 in Supplement 1).^25,30^

### Sensitivity and Scenario Analyses

We conducted deterministic, 1-way sensitivity analyses to examine the outcomes of differences in age at HIV infection by simulating a cohort of Black MSM who acquire HIV at the same age as White MSM (35.5 years). We additionally performed 1-way sensitivity analyses by varying the proportion of MSM who never, formerly, and currently smoke tobacco, using reported 95% CIs of estimated smoking prevalence (eTable 5 in Supplement 1).^25,30^

We examined the implications of equal improvements in care goals and equity-centered goals in 1-way scenario analyses. For equal improvements in care goals, we simulated absolute improvements in receipt of care of 5 or 15 percentage points (vs base case of 10-point increased receipt of care). In additional 1-way scenario analyses, we examined YLG with attaining equity-centered goals compared with different status quo scenarios, which reflect the wide range of current access to care among different regions and demographic groups in the US.^31,32^ We varied the frequency of HIV testing in status quo to every 6 years and compared YLG if annual testing were attained. We next varied the status quo strategy receipt of care (75%-90%) and compared YLG attained with 95% receiving HIV care. We then varied status quo virologic suppression (75%-90%) and compared YLG with 95% virologic suppression*.*

Last, we conducted multiway scenario analyses to estimate YLG with attaining an equitable care continuum in regions with different status quo HIV care continuums (eTable 6 in Supplement 1). We varied status quo receipt of care and virologic suppression (each from 75% to 95%) at 4 status quo HIV testing frequencies: every 6 years, at base case frequency, annually, and every 6 months. We used Excel version 16.0 (Microsoft) to summarize model outcomes. Data were analyzed from July 2021 to October 2023.

## Results

### Base Case

**Figure 1.**
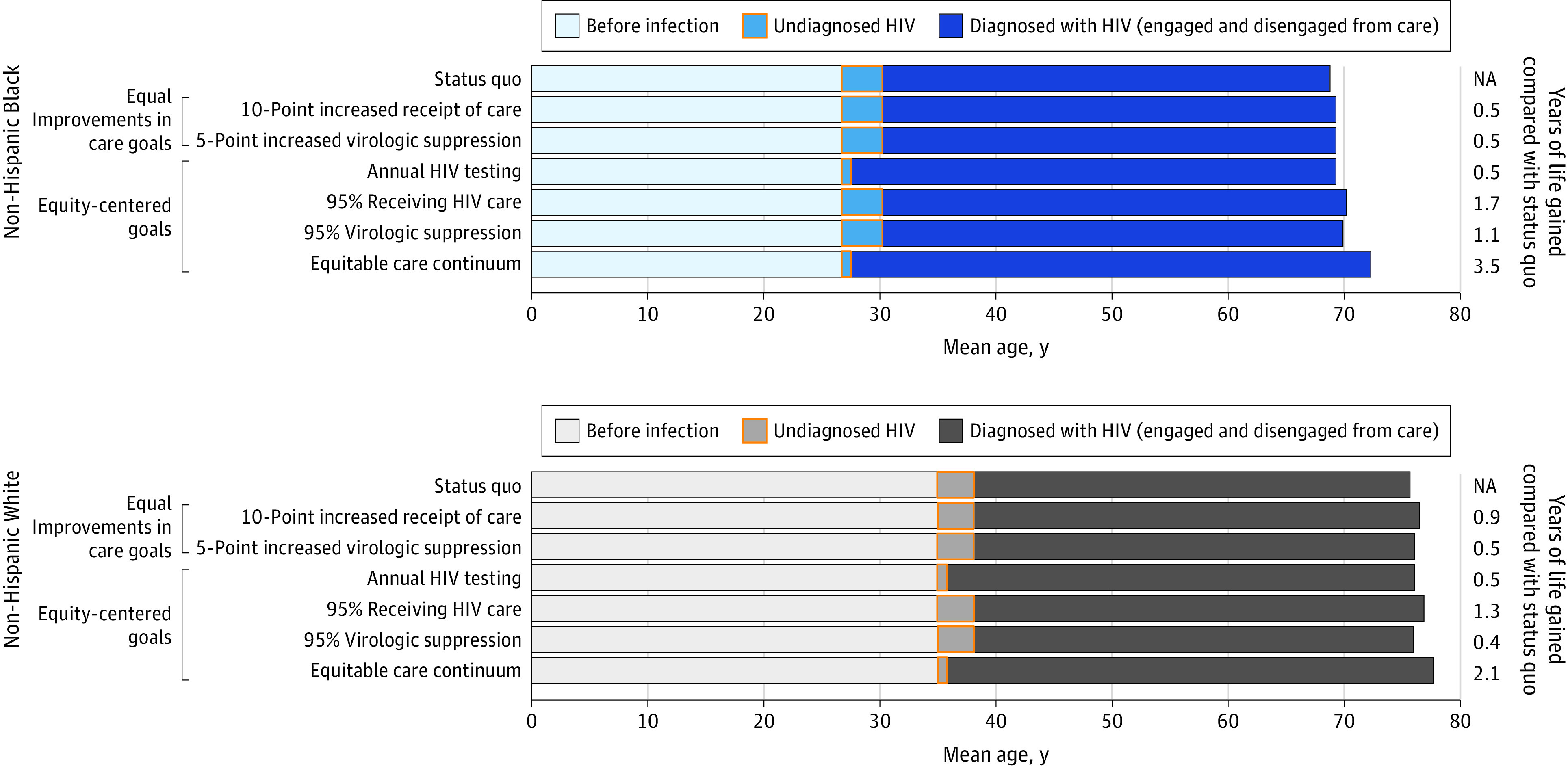
Model-Projected Years of Life Gained With Equal Improvements in Care Goals, Equity-Centered Goals, and an Equitable Care Continuum Among Non-Hispanic Black and White Men Who Have Sex With Men With HIV Compared With Status Quo Figure shows mean age at HIV acquisition, mean age at HIV diagnosis, and mean time lived with diagnosed HIV in the 7 simulated HIV care scenarios. Each bar represents model-projected outcomes for a different HIV care continuum goal. The lighter portions of the bar (left) represent years spent without HIV. The yellow highlighted boxes represent time spent with undiagnosed HIV; the left border of the yellow box represents the mean age at HIV acquisition, and the right border represents the mean age at time of HIV diagnosis. The darker portions of the bars (right) represent time spent with diagnosed HIV, during which the simulated individual could be receiving or disengaged from HIV care.

We projected mean age at death to be 68.8 years among Black MSM and 75.6 years among White MSM with HIV who receive status quo HIV care, a difference of 6.8 years (Figure 1). With the 10-point increased receipt of care goal, Black MSM would gain 0.5 life-years and White MSM would gain 0.9 life-years. The 5-point increased virologic suppression goal would result in 0.5 YLG for Black MSM and 0.5 YLG for White MSM. Equal improvements in care goals would maintain or worsen inequities in age at death between Black MSM and White MSM.

With the first equity-centered goal (annual HIV testing), the mean diagnostic delay would be reduced to 0.9 years for both Black MSM and White MSM. With annual HIV testing, Black MSM and White MSM would gain 0.5 life-years (Figure 1). With 95% receiving HIV care, Black MSM would gain 1.7 life-years, and White MSM would gain 1.3 life-years compared with status quo*.* With 95% virologic suppression, Black MSM would gain 1.1 life-years, and White MSM 0.4 life-years compared with status quo. With attainment of an equitable care continuum, Black MSM would gain 3.5 life-years from status quo, whereas White MSM would gain 2.1 life-years, narrowing the gap in age at death to 5.4 years from 6.8 years.

### One-Way Sensitivity and Scenario Analyses

When simulating a cohort of Black MSM who acquire HIV at a mean (SD) age of 35.5 (16.4) years, age at death would be 71.2 years (White MSM age at death, 75.6 years). When varying smoking prevalence, age at death would range between 68.4 and 69.3 years among Black MSM and between 75.6 and 76.0 years among White MSM. With varying smoking prevalence, model-projected outcomes would change by 0.1 YLG or less compared with the base case. The differences in age at death between Black MSM and White MSM were 7.2 years and 6.7 years under the scenarios of more and less prevalent smoking, respectively. If receipt of care improved by 5 to 15 percentage points in the equal improvements in care goals, Black MSM would experience 0.3 to 0.8 YLG and White MSM 0.4 to 1.3 YLG compared with status quo.

When attaining equity-centered goals with different status quo scenarios, Black MSM would gain 0.8 life-years, and White MSM would gain 0.7 life-years with attainment of the annual HIV testing goal in regions with status quo HIV testing every 6 years. When varying status quo receipt of care (from 90% to 75%), we projected that attaining 95% receiving HIV care would result in 0.5 to 1.3 YLG for Black MSM and 0.5 to 1.7 YLG for White MSM. When varying status quo virologic suppression (from 90% to 75%), we projected that achieving 95% virologic suppression would result in 0.3 to 1.7 YLG for Black MSM and 0.3 to 1.5 YLG for White MSM.

#### Multiway Scenario Analyses

**Figure 2.**
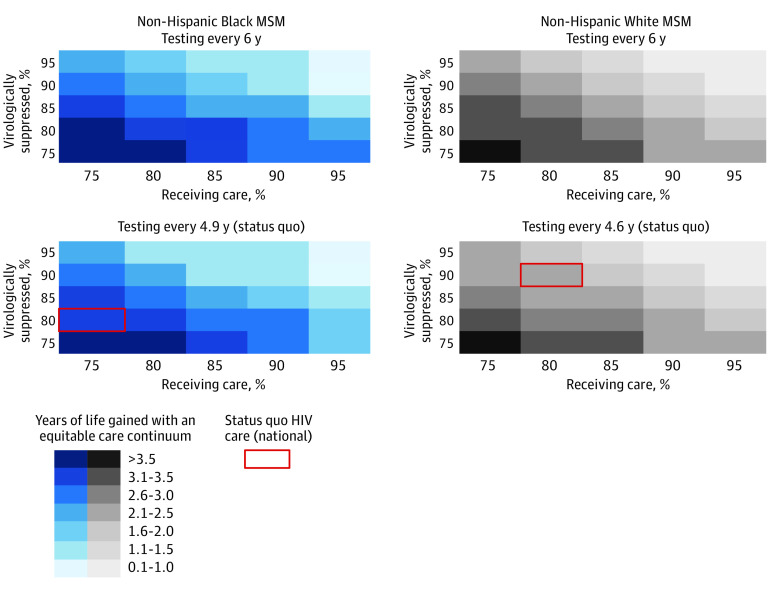
Model-Projected Years of Life Gained With an Equitable Care Continuum of HIV Care Compared With a Range of Status Quo Scenarios, Including Less Frequent HIV Testing Rates We varied the HIV testing frequency (status quo testing [every 4.9 for Black men who have sex with men (MSM) and 4.6 for White MSM] and every 6 years), percentage receiving care (x-axis), and percentage virologically suppressed (y-axis) among non-Hispanic Black MSM (left, blue) and White MSM (right, gray) with HIV in the status quo care continuum. We then projected age at death with the equitable care continuum (annual HIV testing, 95% receiving HIV care, and 95% virologically suppressed) and quantified the years of life gained compared with status quo. Darker shades represent greater potential years of life gained when status quo includes less frequent HIV testing (top rows), lower receipt of care (x-axis, left), or lower virologic suppression (y-axis, bottom). The boxes in yellow represent the base case status quo national HIV care continuum.

**Figure 3.**
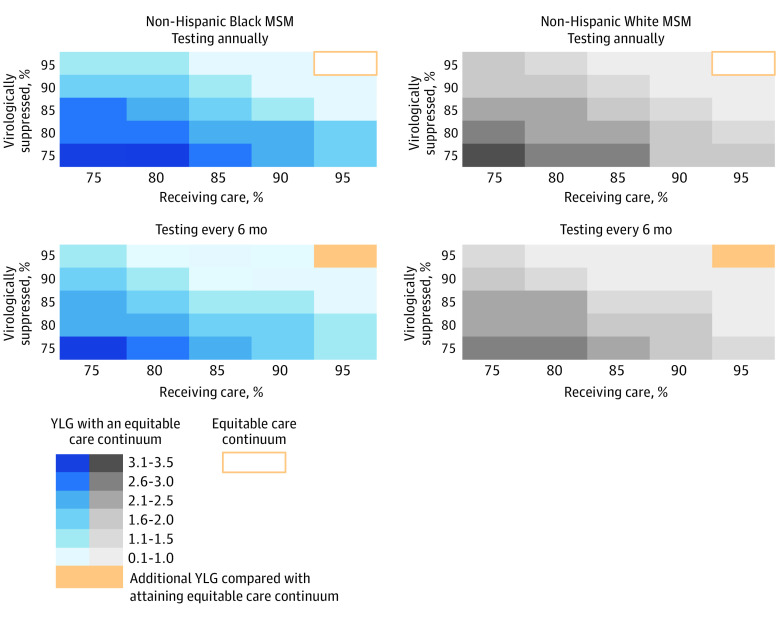
Model-Projected Years of Life Gained With an Equitable Care Continuum of HIV Care Compared With a Range of Status Quo Scenarios With More Frequent HIV Testing Than Current Status Quo We varied the HIV testing frequency (annually and every 6 months), percentage receiving care (x-axis), and percentage virologically suppressed (y-axis) among non-Hispanic Black men who have sex with men (MSM) (left, blue) and White MSM (right, gray) with HIV in status quo care continuum. We then projected age at death with the equitable care continuum (annual HIV testing, 95% receiving HIV care, and 95% virologically suppressed) and quantified the years of life gained compared with status quo. For scenarios in which age at death was greater than age at death under the equitable care continuum scenario, we quantified the additional years of life gained beyond the equitable care continuum. Darker shades represent greater potential years of life gained when status quo includes less frequent HIV testing (top rows), lower receipt of care (x-axis, left), or lower virologic suppression (y-axis, bottom). The solid yellow boxes represent scenarios in which years of life gained would be greater than that projected with the equitable care continuum due to more frequent HIV testing than annually. The box outlined in yellow represents the base case equitable care continuum.

We projected YLG if an equitable care continuum was attained compared with different status quo HIV care continuums. In regions with infrequent HIV testing (every 6 years), projected outcomes with an equitable care continuum would result in 0.7 to 4.2 YLG for Black MSM and 0.5 to 4.0 YLG for White MSM if status quo receipt of care and virologic suppression were both 95% or 75%, respectively (Figure 2). An equitable care continuum would result in 0.6 to 4.0 YLG for Black MSM and 0.5 to 3.7 YLG for White MSM in regions with status quo HIV testing frequency (Black MSM, every 4.9 years; White MSM, every 4.6 years), and receipt of care and virologic suppression were both 95% or 75%, respectively (Figure 2). In regions with annual HIV testing in status quo but 75% receiving care and 75% virologically suppressed, attaining an equitable care continuum would result in up to 3.4 YLG for Black MSM and 3.2 YLG for White MSM (Figure 3). Further improvements in care (HIV testing every 6 months, 95% receiving HIV care, and 95% virologic suppression) would result in an additional 0.2 YLG (Black MSM) and 0.3 YLG (White MSM) compared with an equitable care continuum and annual HIV testing (Figure 3).

## Discussion

We assessed existing inequities across the HIV care continuum and projected the potential outcomes of attaining different goals in the HIV care continuum to address racial inequities, using a decision analytical model. We projected a 6.8-year difference in age at death between Black MSM and White MSM with HIV in the US, according to the current HIV care continuum. Attaining equal improvements in care goals would maintain or worsen existing inequities. In contrast, the equity-centered goal of 95% virologic suppression would result in 0.7 more life-years gained for Black MSM compared with White MSM and thus reduce but not eliminate existing inequities. The greatest potential benefits would occur with an equitable care continuum, particularly among Black MSM, who would gain 3.5 life-years. These model-based results emphasize how an equity-centered approach across the HIV care continuum is critical to improve care outcomes and reduce racial inequities in HIV care.

When examining scenarios that focus on individual steps in the care continuum, we found that improving receipt of care would result in more years of life gained than achieving national goals for improving HIV testing or virologic suppression. These findings are likely due to the larger gap between current and goal levels of receiving HIV care compared with testing and virologic suppression. Additionally, synergies exist in the HIV care continuum; more people can attain virologic suppression if more people receive HIV care.

Although interventions to increase receipt of HIV care will improve clinical outcomes, our findings highlight that a combination of equity-centered strategies is critical. A combination of more frequent HIV testing, improved receipt of care, and increased virologic suppression would result in at least twice the gain in life-years than would occur with achieving an improvement goal in any 1 of the care continuum steps.

To achieve an equitable care continuum, there is a need for investment in and prioritization of evidence-based, multilevel interventions developed to meet the needs of Black MSM in different community contexts and that consider local HIV epidemic dynamics.^33,34,35,36^ For example, it is essential to address structural issues due to health care infrastructure (eg, dearth of HIV practitioners, lack of Medicaid expansion, and medical mistrust) that are associated with a higher burden of HIV in the US South and to develop tailored interventions focused on the community and cultural context for Black MSM residing in the US South. Such interventions are also likely to provide benefits to the community at large by reducing forward transmission of HIV (eg, treatment as prevention) among other benefits.^3,37^

The importance of equity-centered initiatives, with explicit, longitudinal engagement of disproportionately affected communities, has been recognized at both the local and federal level.^38,39^ For example, the National Institutes of Health have funded the E2i Initiative^5,38,40,41^ to develop and more rapidly scale-up interventions among key populations, such as Black MSM. One example is the Health Models Program,^42^ which provided financial incentives, counseling, and patient navigation services to Black MSM with HIV at a total mean cost of $161/person during the 3-year study. Another program has formed close partnerships with Black-owned barbershops to increase access to health information, with the goal of decreasing HIV stigma and increasing linkage and engagement in care.^43^ Recent advances in treatment, particularly long-acting injectable ART formulations, also hold promise to improve virologic suppression but must be implemented using care models that ensure needed access to priority populations despite structural barriers.^23,44^ Most published interventions that focus on improving HIV care continuum outcomes for Black MSM have been individual-level interventions focused on medication adherence; structural interventions that further address multiple steps in the care continuum are needed.^3,5^

Even with attainment of an equitable care continuum, this modeling analysis projects that Black MSM with HIV would still die at a younger age than White MSM with HIV. This finding is in part, but not entirely, due to the earlier mean age at which Black MSM acquire HIV than White MSM. Improving uptake of HIV prevention strategies, such as PrEP, is of critical importance to ending the HIV epidemic and reducing inequities, and equity-centered interventions must also include prevention services for people without HIV, including equitable access to PrEP and harm reduction services.^45^ The gap in life expectancy is also likely due to inequities in non–HIV-related mortality. Black men experience higher rates of chronic health conditions than White men.^46,47,48^ Social and structural barriers drive these health inequities, including unequal access to health care, income and housing inequality, and multilevel stigma, all of which contribute to both HIV-related and non–HIV-related health risks.^7,8^

Our study adds to a growing body of studies that estimate the potential benefits of specific initiatives on inequities along the HIV care continuum.^14^ A recent modeling analysis found that focusing HIV prevention and treatment service distribution to match the racial and ethnic proportion of new HIV diagnoses in 6 US cities would result in greater gains of quality–adjusted life-years than if services were scaled up by an equal absolute amount across racial and ethnic groups.^49^ This study used distributional cost-effectiveness analysis, which provides a methodological framework to assess specific strategies projected to worsen (or reduce) disparities in outcomes between groups when also considering cost.^50^ We recommend that future cost-effectiveness analyses include distributional cost-effectiveness analysis methods to expand the evidence base for strategies that would reduce racial inequities.

### Limitations

There are several limitations to this analysis. Model-projected outcomes are affected by data uncertainty, which we examined with sensitivity and scenario analyses.^51^ Because this analysis focused on individual outcomes for people with HIV, we did not include HIV transmissions or PrEP, which are crucial components in multifaceted strategies to decrease transmissions and end the HIV epidemic.^52,53^ Black MSM and White MSM were the focus of this analysis; further analysis is needed to assess implications for MSM who identify as Hispanic and/or Alaskan Native and Indigenous American, as well as other communities who experience high barriers to care, including women with HIV. This study quantified inequities in age at death and the potential years of life gained if HIV care goals were met; further knowledge of the specific drivers of inequities is needed to develop effective, evidence-based strategies to reduce inequities.

## Conclusions

Equity-centered solutions across the HIV care continuum are critical to mitigate inequities in care among MSM with HIV in the US. Increasing receipt of care and virologic suppression using equity-centered goals would result in more years of life gained for Black MSM compared with White MSM in the US, given the current status quo; this would begin to bring life expectancy among Black MSM closer to parity with White MSM. Our results suggest that equity-centered approaches could be a successful strategy to improve HIV care for MSM. Allocating resources to develop and identify effective equity-centered, evidence-based interventions for Black MSM and implementation strategies to deploy these interventions will be essential to achieve health equity across the HIV care continuum.

## Notes

Supplement 1.
eAppendix

eReferences
**eTable 1.** Comparison of Published Data and CEPAC Model Outcomes for Selected CEPAC Model Parameters of Status Quo HIV Care **eTable 2.** Selected CEPAC Model Input Parameters by HIV Care Scenario **eTable 3.** Comparison of Published Data and CEPAC Model Outcomes During First 5 Years From Initiation of ART for Receipt of Care and Virologic Suppression **eTable 4.** Relative Mortality Ratios by Smoking and Prevalence Among Black and White People With HIV **eTable 5.** Range of Smoking Prevalence Used in Sensitivity Analyses Derived From Mdodo et al **eTable 6.** Current Receipt of Care and Virologic Suppression Levels Reported Among Black MSM and White MSM in Georgia, California, and New York City **eFigure 1.** The HIV Care Continuum Among Non-Hispanic Black and White MSM With HIV in the US With Status Quo and Equal Improvements in Care Goals **eFigure 2.** The HIV Care Continuum Among Non-Hispanic Black and White MSM With HIV in the US With Equity-Centered Goals, and Equitable Care Continuum **eFigure 3.** Model Schematic

Supplement 2.
Data Sharing Statement
