# Supplementary material for: Projected Life Expectancy Gains From Improvements in HIV Care in Black and White Men Who Have Sex With Men
Source: JAMA Netw Open. 2023 Nov 28;6(11):e2344385. doi: 10.1001/jamanetworkopen.2023.44385 (PMC10685884; doi:10.1001/jamanetworkopen.2023.44385)
Supplement: Supplement 1. — eAppendix eReferences eTable 1. Comparison of Published Data and CEPAC Model Outcomes for Selected CEPAC Model Parameters of Status Quo HIV Care eTable 2. Selected CEPAC Model Input Parameters by HIV Care Scenario eTable 3. Comparison of Published Data and CEPAC Model Outcomes During First 5 Years From Initiation of ART for Receipt of Care and Virologic Suppression eTable 4. Relative Mortality Ratios by Smoking and Prevalence Among Black and White People With HIV eTable 5. Range of Smoking Prevalence Used in Sensitivity Analyses Derived From Mdodo et al eTable 6. Current Receipt of Care and Virologic Suppression Levels Reported Among Black MSM and White MSM in Georgia, California, and New York City eFigure 1. The HIV Care Continuum Among Non-Hispanic Black and White MSM With HIV in the US With Status Quo and Equal Improvements in Care Goals eFigure 2. The HIV Care Continuum Among Non-Hispanic Black and White MSM With HIV in the US With Equity-Centered Goals, and Equitable Care Continuum eFigure 3. Model Schematic [file jamanetwopen-e2344385-s001.pdf]

Supplemental Online Content

Rich KM, Pandya A, Chiosi JJ, et al. Projected life expectancy gains from improvements in HIV care in men who have sex with men. *JAMA Netw Open*. 2023;6(11):e2344385. doi:10.1001/jamanetworkopen.2023.44385

eAppendix
eReferences
eTable 1. Comparison of Published Data and CEPAC Model Outcomes for Selected CEPAC Model Parameters of Status Quo HIV Care
eTable 2. Selected CEPAC Model Input Parameters by HIV Care Scenario
eTable 3. Comparison of Published Data and CEPAC Model Outcomes During First 5 Years From Initiation of ART for Receipt of Care and Virologic Suppression
eTable 4. Relative Mortality Ratios by Smoking and Prevalance Among Black and White People With HIV
eTable 5. Range of Smoking Prevalence Used in Sensitivity Analyses Derived From Mdodo et al
eTable 6. Current Receipt of Care and Virologic Suppression Levels Reported Among Black MSM and White MSM in Georgia, California, and New York City
eFigure 1. The HIV Care Continuum Among Non-Hispanic Black and White MSM With HIV in the US With Status Quo and Equal Improvements in Care Goals
eFigure 2. The HIV Care Continuum Among Non-Hispanic Black and White MSM With HIV in the US With Equity-Centered Goals, and Equitable Care Continuum
eFigure 3. Model Schematic

This supplemental material has been provided by the authors to give readers additional information about their work.

© 2023 Rich KM et al. *JAMA Network Open.*

INTRODUCTION

In this supplemental appendix, we provide further details of the simulation modeling methods and derivation of input parameters for simulated cohorts of non-Hispanic Black men who have sex with men (MSM) and non-Hispanic White MSM.

METHODS

Full details of the model, which is coded in C++**,** are available at https://www.massgeneral.org/medicine/mpec/research/cpac-model**.**

Age at model start

All simulated individuals enter the model at birth and without HIV. HIV acquisition is determined by a race-specific monthly risk starting at age 15y. For a schematic of the model refer to eFigure 3.

Mean age at time of HIV diagnosis

We estimated the mean age at time of diagnosis using 2021 data reported by the US Centers of Disease Control and Prevention (CDC).[1] We used data from the transmission category, 'male-to-male sexual contact,' among non-Hispanic Black men (for Black MSM) and non-Hispanic White men (for White MSM). From the grouped data, we estimated mean age and standard deviation at diagnosis, using the frequency in each group and the mean age of each stratum. We calculated mean age at diagnosis to be 30.4 years (SD: 10.8 years) among Black MSM and 38.8 years (SD: 13.6 years) for White MSM.[1]

© 2023 Rich KM et al. *JAMA Network Open.*

CD4 count at diagnosis

We calculated the mean CD4 count (cells/µL) at the time of diagnosis from 2021 data from the CDC HIV Surveillance Reports, which reported the number of people diagnosed with HIV stratified by CD4 stage at the time of diagnosis.[2] For Stage 1 diagnoses (reported by the CDC as $\geq$500 cells/µL), we assumed the CD4 range to be 500-750 cells/µL. Diagnoses categorized as Stage 0 or unknown were excluded from calculations. From these grouped data, we estimated mean CD4 count and standard deviation, using the frequency of cases within each stratum and the mean CD4 count of each stratum. We estimated the mean CD4 count at diagnosis: 380 cells/µL (SD: 195) among Black MSM and 373 cells/µL (SD: 209) among White MSM (eTable 2).

Mean time from infection to diagnosis ("diagnostic delay")

In the CEPAC model, all simulated individuals have a monthly probability of HIV diagnosis following HIV infection, either from screening or from diagnostic testing after an opportunistic infection (OI). To simulate this probability of HIV diagnosis, we first estimated mean time from infection to diagnosis, using a previously published CD4 depletion model.[3] With this model, we back-calculated the expected time of undiagnosed (and untreated) HIV that would account for the CD4 count observed at diagnosis, using the rate of CD4 decline. We estimated that the time from infection until diagnosis was 3.4 years among Black MSM and 3.3 years among White MSM. We then calibrated the CEPAC monthly probability of HIV diagnosis for Black MSM and for White MSM, independently, to match the calculated diagnostic delay (eTable 2).

© 2023 Rich KM et al. *JAMA Network Open.*

Mean age at time of infection

We calculated the mean age at time of HIV infection for Black MSM and White MSM, separately, with the following formula:

$$\text{Estimated age of infection} = \text{Age at time of HIV diagnosis} - \text{diagnostic delay}$$

Then, we calibrated the monthly probability of HIV infection for Black MSM and White MSM in the CEPAC model to simulate the estimated average age of HIV infection starting from age 15y: 0.0063 (Black MSM) and 0.0027 (White MSM).

Receipt of HIV care, ART adherence, & virologic suppression

In CEPAC, each simulated individual is assigned a propensity to respond (PTR) value, drawn from a normal distribution.[4] The PTR value determines a simulated individual's adherence to ART, monthly probability of disengagement in care, probability of virologic suppression within 6 months of initiating ART, as well as the monthly probability of virologic non-suppression occurring after initial suppression; higher ART adherence results in a greater probability of attaining and sustaining virologic suppression.

Propensity to respond (PTR) & loss to follow-up

We calibrated the PTR distribution and parameterized loss to follow-up estimates for Black MSM and White MSM so that our simulated estimates of: (1) mean percent of time receiving HIV care and (2) mean percent of time with virologic suppression for 5 years following diagnosis matched receipt of care estimates from CDC 2021 data and estimates from NA-ACCORD (2004 – 2014).[2,5]

© 2023 Rich KM et al. *JAMA Network Open.*

Receipt of care

We first calibrated the PTR distribution and loss to follow-up estimates so that model projections of total person-time spent in HIV care after HIV diagnosis for 5 years matched cross-sectional data in 2021 CDC HIV Surveillance data of the percent of individuals who received HIV care ($\geq$1 viral load or CD4 count measurement in 2021) among MSM with diagnosed HIV.[2] We then compared model-projected outcomes of the percent of total person-time MSM with HIV were receiving care for first five years from diagnosis with estimates from NA-ACCORD (2004 – 2014).[5]

Virologic suppression

We then further calibrated the PTR distribution and LTFU function so that the model projections of percent of time spent virologically suppressed out of total time spent on ART over the first 5 years from diagnosis matched 2021 CDC HIV Surveillance data of percent of individuals with virologic suppression (last recorded viral load (VL) <200 copies/mL) out of those who had received care during 2021 ($\geq$1 viral load or CD4 count measurement in 2021).[2]

Final calibration

Final inputs for PTR and loss to follow-up parameters were the combination that resulted in the least sum of squares as follows (eTable 3 & eTable 4):

$$Sum\ of\ squares\ (with\ given\ input\ of\ PTR\ and\ LTFU\ function) =$$

$$(Engaged\ in\ Care\ Calibration\ Target - Engaged\ in\ Care\ Model\ ouput)^2$$

$$+$$

$$(Virologic\ Suppression\ Calibration\ Target - Virologic\ Suppression\ Model\ Output\ )^2$$

© 2023 Rich KM et al. *JAMA Network Open.*

Re-engagement in care

Simulated individuals who have been diagnosed with HIV and disengaged from care have a monthly probability of returning to care (RTC). Black MSM and White MSM cohorts each had a distinct RTC monthly probability, stratified by race/ethnicity x geography x transmission group x sex.[6] To calculate RTC probabilities for Black MSM and White MSM, we used the paper's probabilities for Black MSM and White MSM and weighted the estimates to match geographical distribution of MSM diagnosed with HIV using CDC Atlas Plus 2019 data. Monthly RTC probability was 4.4% for Black MSM and 3.2% for White MSM, which corresponds to a mean of 1.9 years out of care per episode for Black MSM and 2.6 years out of care per episode for White MSM, assuming a constant monthly probability of RTC.

Non-HIV-related mortality

To allow for a simulation of individuals who acquire HIV and to reduce the risk of competing mortality risk among simulated individuals who have not yet acquired HIV, non-HIV-related mortality was zero until the mean age of HIV infection for Black MSM and White MSM, respectively.

Non-HIV-related mortality was calculated using race/ethnicity-specific 2019 CDC life tables (National Vital Statistics) for Black men and White men.[7] The national life tables were adjusted for increased tobacco smoking-related mortality among Black MSM and White MSM, given the higher prevalence of tobacco smoking among these groups compared with the general adult male population. We adjusted mortality attributable to tobacco smoking among MSM with HIV using race-specific prevalence estimates of tobacco smoking status among people with HIV (PWH):

© 2023 Rich KM et al. *JAMA Network Open.*

formerly smoked (Black PWH, 15.5%; White PWH, 27.2%), currently smoke (Black PWH, 43.9%; White PWH, 43.8%), and never smoked (Black PWH, 40.6%; White PWH, 29.0%), using previously published relative mortality ratios for never smoked (mortality risk ratio [RR], 1.0), formerly smoked (RR, 1.5), and currently smoking (RR, 2.8) (eTable 5).[8–11]

Baseline life tables: 2019 CDC life tables for non-Hispanic Black men (for Black MSM) and non-Hispanic White men (for White MSM)

1. **Converted national life tables to life tables for 'Never Smoked:'** Using an estimated prevalence of smoking in the general population,[12] we converted national race- and sex-specific life tables to a life table corresponding to a cohort of individuals who have 'Never Smoked,' using methods previously outlined.[8] The mortality rate of the general population and never smokers remain the same until age 40y.
2. **Final life table:** Using the 'Never Smoker' life tables, we next calculated cohort life tables for Black MSM and White MSM with HIV, respectively. We did this by using data on smoking prevalence among Black MSM and White MSM with HIV and relative mortality ratios.[8,9] Individuals who had formerly smoked were estimated to have a relative mortality ratio of 1.5 compared with never smokers; individuals currently smoking were estimated to have a relative mortality risk of 2.8 compared with never smokers.

© 2023 Rich KM et al. *JAMA Network Open.*

ART regimens

We used ART regimens recommended by the US Department of Health and Human Service (DHHS) Guidelines for the Use of Antiretroviral Agents in Adults and Adolescents living with HIV.[13] All simulated individuals with diagnosed HIV initiate ART with an integrase strand transfer inhibitor (INSTI)-based regimen as per DHHS guidelines.[13,14] Probabilities of initial virologic suppression and subsequent virologic non-suppression are based on clinical trial data for each respective ART line.[14] Individuals with diagnosed HIV who do not attain virologic suppression on an INSTI-based regimen and who remain in care switch to another guideline-based alternative ART regimen. Among individuals with ART adherence of 95% or greater on an INSTI-based regimen, 96.4% achieve virologic suppression at 12 months, followed by a small monthly probability of loss of suppression (0.19%).[15–17]

Simulation of HIV Care Continuum Goals

Equal Improvements in Care Goals

To simulate the *10-Point Increased Receipt of Care* goal, we increased receipt of care among Black MSM and White MSM diagnosed with HIV by 10 percentage points, with all other aspects of HIV care remaining at *Status Quo*. While the proportion of MSM receiving HIV care who attain virologic suppression remains at *Status Quo* levels, more MSM with HIV attain virologic suppression because more MSM with diagnosed HIV are receiving care. With the *5-Point Increased Virologic Suppression* goal, we increased virologic suppression among simulated Black MSM and White MSM receiving HIV care by 5 percentage points.

© 2023 Rich KM et al. *JAMA Network Open.*

Equity-Centered Goals

To simulate *Annual HIV Testing,* we calibrated input parameters so that all MSM received an HIV diagnosis within one year of HIV infection. A secondary impact of *Annual HIV Testing* is that the total number of Black MSM and White MSM receive HIV care and virologically suppressed increases; the same proportion of Black MSM and White MSM with diagnosed HIV, receive HIV care and attain virologic suppression as *Status Quo* levels, but more MSM with HIV are diagnosed with *Annual HIV Testing*.

To simulate *95% Receiving HIV Care,* we calibrated input parameters so that 95% of total person time of MSM with diagnosed HIV was spent receiving HIV care for the first 5 years following HIV diagnosis. While the proportion of virologic suppression among MSM receiving care remains at *Status Quo* levels, a secondary impact of *95% Receiving HIV Care* is that more MSM with HIV attain virologic suppression because more MSM with diagnosed HIV are receiving care.

In *95% Virologic Suppression*, we calibrated the model so that 95% of person time among MSM receipt of care is with virologic suppression for the first 5 years from diagnosis. HIV testing frequency and the proportion of time spent receiving care remain at *Status Quo* levels.

Equitable Care Continuum Scenario

To simulate an *Equitable Care Continuum,* we calibrated input parameters for Black MSM and White MSM to attain a combination of the Equity-Centered Goals: annual HIV testing, 95% receipt of care, and 95% virologic suppression. In this scenario, all MSM are diagnosed within

© 2023 Rich KM et al. *JAMA Network Open.*

one year of HIV acquisition, 95% of total person time among MSM with diagnosed HIV is spent in care for the first five years following HIV diagnosis, and 95% of total person time among MSM in care is with virologic suppression for the first five years following HIV diagnosis. In this scenario, Black MSM and White MSM attain equitable HIV diagnosis rates, receipt of HIV care**,** and virologic suppression.

Sensitivity and Scenario Analyses

Age at HIV Acquisiton

We performed one-way sensitivty analyses on age at HIV acquisiton by simulated a cohort of Black MSM who acquired HIV at the same age as White MSM.

Smoking prevalance sensitivity analysis

We performed one-way sensitivity analysis, using the upper and lower bounds of smoking prevalence estimates among Black and White MSM (eTable 6).[9]

Testing frequency scenario analyses

To perform scenario analysis to assess uncertainity around testing frequency estimates, we varied the rate of HIV testing to simulate HIV screening every six years, every year (i.e. annually), and every six months.

Receipt of care and virologic suppression scenario analsyes

We examined the variation in the potential impact of Equity Centered Goals when the *Status Quo* scenario differed, given the wide range of current HIV care continuums between regions

© 2023 Rich KM et al. *JAMA Network Open.*

and demographic groups **(**eTable 7). To perform scenario analyses that assess the variation in *Status Quo* receipt of care and virologic suppression, we performed scenario analyses that varied receipt of HIV care (75% to 95%) and virologic suppression (75% to 95%). To complete these simulation runs, the CEPAC model input parameters of PTR and the loss to folllow-up parameters were calibrated to match desired receipt of care and virologic suppression output levels.

© 2023 Rich KM et al. *JAMA Network Open.*

eReferences

1. Centers for Disease Control and Prevention. *Diagnoses of HIV Infection in the United States and Dependent Areas, 2021*.; 2023. Accessed September 22, 2023. https://www.cdc.gov/hiv/library/reports/hiv-surveillance/vol-34/index.html

2. Centers for Disease Control and Prevention. *Monitoring Selected National HIV Prevention and Care Objectives by Using HIV Surveillance Data—United States and 6 Dependent Areas, 2021*.; 2023. Accessed September 22, 2023. https://www.cdc.gov/hiv/library/reports/hiv-surveillance/vol-28-no-4/index.html

3. Song R, Hall HI, Green TA, Szwarcwald CL, Pantazis N. Using CD4 data to estimate HIV incidence, prevalence, and percent of undiagnosed infections in the United States. *J Acquir Immune Defic Syndr*. 2017;74(1):3-9. doi:10.1097/QAI.0000000000001151

4. Ross EL, Weinstein MC, Schackman BR, et al. The clinical role and cost-effectiveness of long-acting antiretroviral therapy. *Clin Infect Dis*. 2015;60(7):1102-1110. doi:10.1093/cid/ciu1159

5. Desir FA, Lesko CR, Moore RD, et al. One size fits (n)one: The influence of sex, age, and sexual Human Immunodeficiency Virus (HIV) acquisition risk on racial/ethnic disparities in the HIV care continuum in the United States. *Clin Infect Dis*. 2019;68(5):795-802. doi:10.1093/cid/ciy556

6. Wang L, Krebs E, Min JE, et al. Combined estimation of disease progression and retention on antiretroviral therapy among treated individuals with HIV in the USA: A modelling study. *Lancet HIV*. 2019;6(8):e531-e539. doi:10.1016/S2352-3018(19)30148-1

7. Arias E, Xu JQ. *United States Life Tables, 2019. National Vital Statistics Reports*.; 2021.

8. Reddy KP, Parker RA, Losina E, et al. Impact of cigarette smoking and smoking cessation on life expectancy among people with HIV: A US-based modeling study. *J Infect Dis*. 2016;214(11):1672-1681. doi:10.1093/infdis/jiw430

9. Mdodo R, Frazier EL, Dube SR, et al. Cigarette smoking prevalence among adults with HIV compared with the general adult population in the United States. *Ann Intern Med*. 2015;162(5):335-344. doi:10.7326/M14-0954

10. Jha P, Ramasundarahettige C, Landsman V, et al. 21st-century hazards of smoking and benefits of cessation in the United States. *N Engl J Med*. 2013;368(4):341-350. doi:10.1056/NEJMsa1211128

11. Thun MJ, Carter BD, Feskanich D, et al. 50-year trends in smoking-related mortality in the United States. *N Engl J Med*. 2013;368(4):351-364. doi:10.1056/NEJMsa1211127

© 2023 Rich KM et al. *JAMA Network Open.*

12. Frazier EL, Sutton MY, Brooks JT, Shouse RL, Weiser J. Trends in cigarette smoking among adults with HIV compared with the general adult population, United States - 2009-2014. *Prev Med*. 2018;111:231-234. doi:10.1016/j.ypmed.2018.03.007

13. Panel on Antiretroviral Guidelines for Adults and Adolescents. Guidelines for the use of antiretroviral agents in adults and adolescents with HIV. Published January 20, 2022. Accessed May 8, 2023. https://clinicalinfo.hiv.gov/en/guidelines/hiv-clinical-guidelines-adult-and-adolescent-arv/whats-new

14. Hyle EP, Kasaie P, Schwamm E, et al. A growing number of men who have sex with men aging with HIV (2021-2031): A comparison of two microsimulation models. *J Infect Dis*. Published online December 1, 2022:jiac473. doi:10.1093/infdis/jiac473

15. Walmsley SL, Antela A, Clumeck N, et al. Dolutegravir plus abacavir-lamivudine for the treatment of HIV-1 infection. *N Engl J Med*. 2013;369(19):1807-1818. doi:10.1056/NEJMoa1215541

16. Sax PE, Pozniak A, Montes ML, et al. Coformulated bictegravir, emtricitabine, and tenofovir alafenamide versus dolutegravir with emtricitabine and tenofovir alafenamide, for initial treatment of HIV-1 infection (GS-US-380-1490): A randomised, double-blind, multicentre, phase 3, non-inferiority trial. *Lancet*. 2017;390(10107):2073-2082. doi:10.1016/S0140-6736(17)32340-1

17. Gallant J, Lazzarin A, Mills A, et al. Bictegravir, emtricitabine, and tenofovir alafenamide versus dolutegravir, abacavir, and lamivudine for initial treatment of HIV-1 infection (GS-US-380-1489): A double-blind, multicentre, phase 3, randomised controlled non-inferiority trial. *Lancet*. 2017;390(10107):2063-2072. doi:10.1016/S0140-6736(17)32299-7

18. Husereau D, Drummond M, Augustovski F, et al. Consolidated Health Economic Evaluation Reporting Standards (CHEERS) 2022 Explanation and Elaboration: A Report of the ISPOR CHEERS II Good Practices Task Force. *Value Health*. 2022;25(1):10-31. doi:10.1016/j.jval.2021.10.008

19. Centers for Disease Control and Prevention. Monitoring selected national HIV prevention and care objectives by using HIV surveillance data—United States and 6 dependent areas, 2019. Published May 2021. Accessed May 6, 2023. https://www.cdc.gov/hiv/library/reports/hiv-surveillance/vol-26-no-2/index.html

20. Georgia Department of Public Health. Georgia HIV Surveillance Data. Accessed May 8, 2023. https://dph.georgia.gov/epidemiology/georgias-hivaids-epidemiology-section/georgia-hiv-surveillance-data

21. Centers for Disease Control and Prevention. NCHHSTP AtlasPlus. Accessed May 8, 2023. https://gis.cdc.gov/grasp/nchhstpatlas/main.html

22. New York City Department of Health and Mental Hygiene. *HIV among Men Who Have Sex with Men (MSM) in New York City, 2018*. HIV Epidemiology Program; 2019. https://www1.nyc.gov/assets/doh/downloads/pdf/dires/hiv-aids-in-msm.pdf

© 2023 Rich KM et al. *JAMA Network Open.*

eTable 1. Comparison of Published Data and CEPAC Model Outcomes for Selected CEPAC Model Parameters of Status Quo HIV Care

CEPAC Model Parameter	Published Data (Calibration Target)	Source	CEPAC Model Outcome
	**Non-Hispanic Black MSM with HIV**		
Mean age at HIV infection, years (SD)	27.0 (10.8)	Derived from [1–3]	26.9 (11.2)
Mean age at diagnosis, years (SD)	30.4 (10.8)	Derived from [1]	30.3 (11.3)
Mean time from infection to diagnosis, years	3.4 (95% CI: 3.23 – 3.60)	Derived from [1,3]	3.4 (SD: 3.1)
Mean CD4 count at diagnosis, cells/μL	380 (195)	Derived from [2]	388
	**Non-Hispanic White MSM with HIV**		
Mean age at HIV infection (SD)	35.5 (13.6)	Derived from [1–3]	35.4 (17.1)
Mean age at diagnosis, years (SD)	38.8 (13.6)	Derived from [1]	38.7 (16.6)
Mean time from infection to diagnosis (SD)	3.3 (95% CI: 2.87 – 3.22)	Derived from [1,3]	3.3 (SD: 2.8)
Mean CD4 count at diagnosis, cells/μL (SD)	373(209)	Derived from [2]	394

Abbreviations: CEPAC: Cost-effectiveness of Preventing AIDS Complications SD: standard deviation

© 2023 Rich KM et al. *JAMA Network Open.*

eTable 2. Selected CEPAC Model Input Parameters by HIV Care Scenario

**CEPAC Model Parameter**	**HIV Care Scenario**						
	Status Quo	**10-pt Increased Receipt of Care**	**5-pt Increased Virologic Suppression**	**Annual HIV Testing**	**95% Receipt of Care**	**95% Virologic Suppression**	***Equitable Care Continuum***
	**Non-Hispanic Black MSM**						
Propensity to Respond[1]	1.70	1.60	1.80	1.70	1.80	2.80	2.80
Monthly Probability of LTFU if Adherence <50%[2]	0.050	0.020	0.040	0.050	0.005	0.130	0.005
Monthly Testing Probability	0.017	0.017	0.017	0.080	0.017	0.017	0.080
	**Non-Hispanic White MSM**						
Propensity to Respond[1]	2.35	2.40	2.80	2.35	2.35	2.90	2.90
Monthly Probability of LTFU if Adherence <50%[2]	0.060	0.020	0.080	0.060	0.006	0.100	0.006
Monthly Testing Probability	0.018	0.018	0.018	0.080	0.018	0.018	0.080

1. Standard deviation of PTR = 1.5 across all scenarios
2. The probability of LTFU was modeled as a function of ART adherence. All simulated individuals with an adherence rate of ≥98.5% had a monthly LTFU probability of 0.0001.

Abbreviations: CEPAC: Cost-effectiveness of Preventing AIDS Complications, LTFU: lost to follow-up, MSM: men who have sex with men, SD: standard deviation

© 2023 Rich KM et al. *JAMA Network Open.*

eTable 3. Comparison of Published Data and CEPAC Model Outcomes During First 5 Years From Initiation of ART for Receipt of Care and Virologic Suppression

	Published Data (Calibration Target)				CEPAC Model Outcomes	
**Simulated Scenario**	**% receipt of care**	**Source**	**% VS**	**Source**	**% receipt of care**	**% VS**
	**Non-Hispanic Black MSM**					
Status Quo	75.3%	[2,5]	83.6%	[1]	75.0%	82.9%
10-point Increased Receipt of Care	85.3%	n/a	83.6%	[2]	86.5%	79.5%
5-point Increased Virologic Suppression	75.3%	[2,5]	88.6%	n/a	72.1%	87.8%
Annual HIV Testing	75.3%	[2,5]	83.6%	[2]	75.0%	82.9%
95% Receipt of Care	95.0%	n/a	83.6%	[2]	96.2%	81.5%
95% Virologic Suppression	75.3%	[2,5]	95.0%	n/a	75.0%	95.4%
Equitable Care Continuum	95.0%	n/a	95.0%	n/a	98.0%	93.0%
	**Non-Hispanic White MSM**					
Status Quo	80.3%	[2,5]	92.4%	[1]	78.6%	91.4%
10-point Receipt of Care	90.3%	n/a	92.4%	[2]	90.6%	90.4%
5-point Increased Virologic Suppression	80.3%	[2,5]	97.4%	n/a	80.2%	95.0%
Annual HIV Testing	80.3%	[2,5]	92.4%	[2]	78.6%	91.4%
95% Receipt of Care	95.0%	n/a	92.4%	[2]	96.6%	88.9%
95% Virologic Suppression	80.3%	[2,5]	95.0%	n/a	78.7%	95.7%
Equitable Care Continuum	95.0%	n/a	95.0%	n/a	97.6%	93.8%

Abbreviations: MSM: men who have sex with men, VS: virologically suppressed

© 2023 Rich KM et al. *JAMA Network Open.*

eTable 4. Relative Mortality Ratios by Smoking and Prevalance Among Black and White People With HIV

Smoking History	Relative Mortality Ratio		Smoking Prevalence		
	Estimate	Source	Non-Hispanic Black	Non-Hispanic White	Source
Currently Smoking	2.8	[8,10,11]	43.9%	43.8%	[9]
Formerly Smoked	1.5	[8]	15.5%	27.2%	[9]
Never Smoked	1 (Reference)	[8]	40.6%	29.0%	[9]

© 2023 Rich KM et al. *JAMA Network Open.*

eTable 5. Range of Smoking Prevalence Used in Sensitivity Analyses Derived From Mdodo et al[9]

Model Cohort	Lower Bound of Current Smoking Prevalence Estimate			Upper Bound of Current Smoking Prevalence Estimate		
	**Currently Smoking**	**Formerly Smoked**	**Never Smoked**	**Currently Smoking**	**Formerly Smoked**	**Never Smoked**
Black MSM	39.6%	13.5%	46.9%	48.2%	17.6%	34.2%
White MSM	40.6%	24.4%	35.0%	47.1%	29.9%	23.0%

Abbreviations: MSM: men who have sex with men.

© 2023 Rich KM et al. *JAMA Network Open.*

eTable 7. Current Receipt of Care and Virologic Suppression Levels Reported Among Black MSM and White MSM in Georgia, California, and New York City

	**Black MSM**		**White MSM**		
	**% Receipt of care of MSM diagnosed with HIV**	**% Virologically suppressed of MSM in care**	**% Receipt of care of MSM diagnosed with HIV**	**% Virologically suppressed of MSM in care**	**Data Source**
Georgia	75	78	77	90	[20,21]
California	73	82	80	92	[21]
New York City	92	85	94	96	[22]

Abbreviations: MSM: men who have sex with men.

Figure Legends

eFigure 1. The HIV care continuum among non-Hispanic Black and White MSM with HIV in the US with *Status Quo* (Panel 1) and Equal Improvements in Care Goals (Panels 2-3). We display the current *Status Quo* HIV care continuum (Panel 1); a *10-Point Increased Receipt of Care* (Panel 2); a *5-Point Increased Virologic Suppression* (Panel 3). Dark blue bars represent Black MSM with HIV, grey bars represent White MSM with HIV. Yellow solid arrows represent the magnitude of direct improvement in care for each simulated scenario. Yellow, striped bars represent the secondary impact along the HIV care continuum that would result from attaining the simulated care goal. Abbreviations: Dx: diagnosed, MSM: men who have sex with men, VS: virologic suppression.

eFigure 2. The HIV care continuum among non-Hispanic Black and White MSM with HIV in the US with Equity-Centered Goals (Panels 1-3), and *Equitable Care Continuum* (Panel 4*).* We display *Annual HIV Testing* (Panel 1); *95% Receiving HIV Care* (Panel 2); *95% Virologic Suppression* (Panel 3); and *Equitable Care Continuum* (Panel 4). Dark blue bars represent Black MSM with HIV, grey bars represent White MSM with HIV. Yellow solid arrows represent the magnitude of direct improvement in care for each simulated scenario. Yellow, striped bars represent the secondary impact along the HIV care continuum that would result from attaining the simulated care goal. Abbreviations: Dx: diagnosed, MSM: men who have sex with men, VS: virologic suppression.

© 2023 Rich KM et al. *JAMA Network Open.*

eFigure 3. Model Schematic.

This schematic depicts the trajectory of a simulated person in the CEPAC model. After model start, MSM experience a monthly probability of HIV infection starting at age 15y. When an MSM has acquired HIV, they experience a monthly probability of diagnosis. After diagnosis, MSM can be linked to care and offered ART, receive care, disengage from care, and re-engage in care. All MSM are simulated until death. In the figure: a: average time to infection from model start; b: average time from infection to diagnosis; c: probability of linkage to care following diagnosis; d: monthly probability of lost to follow-up; e: monthly probability of re-engaging with HIV care; f: HIV-related mortality; g: non-HIV related mortality; *f*(h): functions which link ART adherence levels and loss to follow-up probability. Abbreviations: ART: antiretroviral treatment, CEPAC: Cost-Effectiveness of Preventing AIDS Complications, MSM: men who have sex with men. OI: opportunistic infection.

© 2023 Rich KM et al. *JAMA Network Open.*

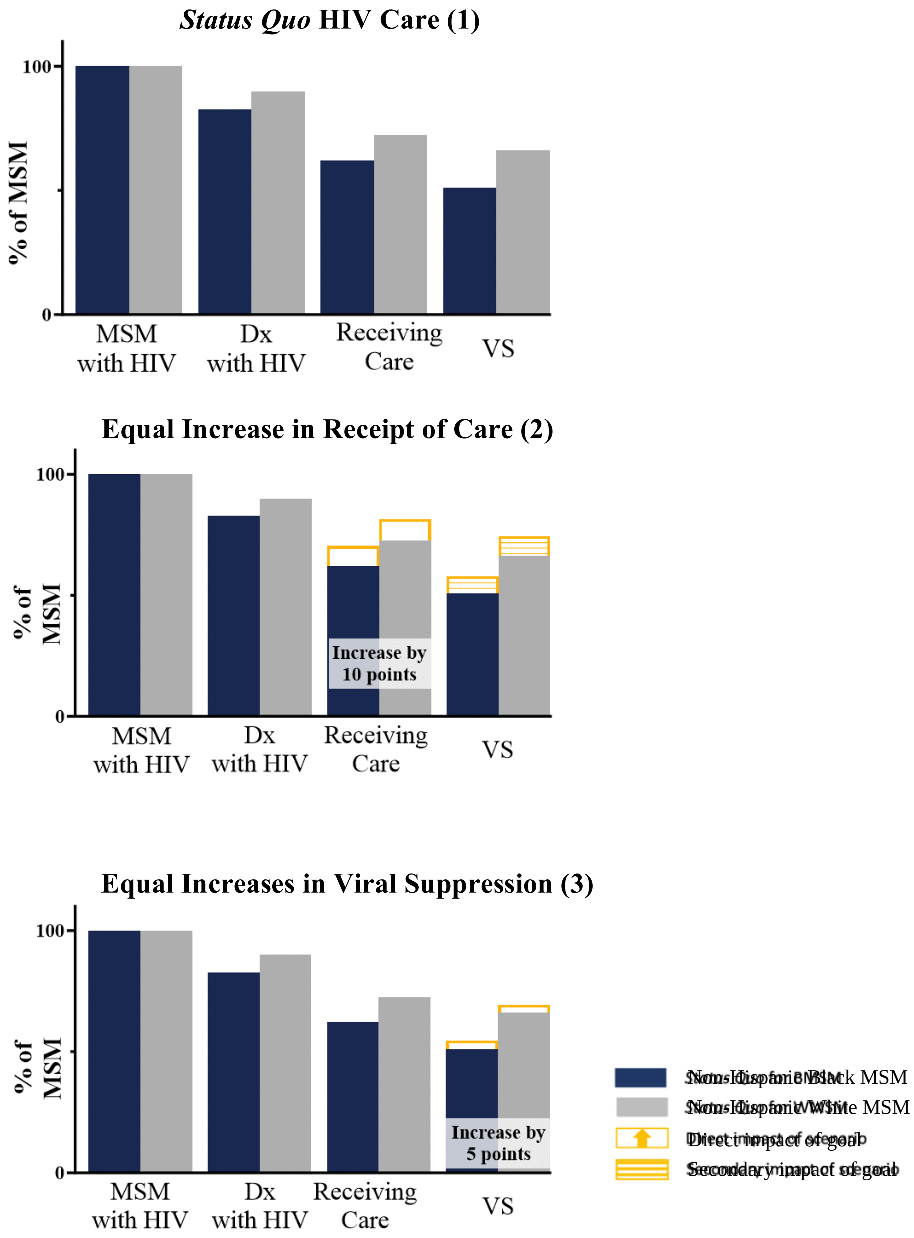

eFigure 1. The HIV Care Continuum Among Non-Hispanic Black and White MSM With HIV in the US With Status Quo and Equal Improvements in Care Goals

© 2023 Rich KM et al. *JAMA Network Open.*

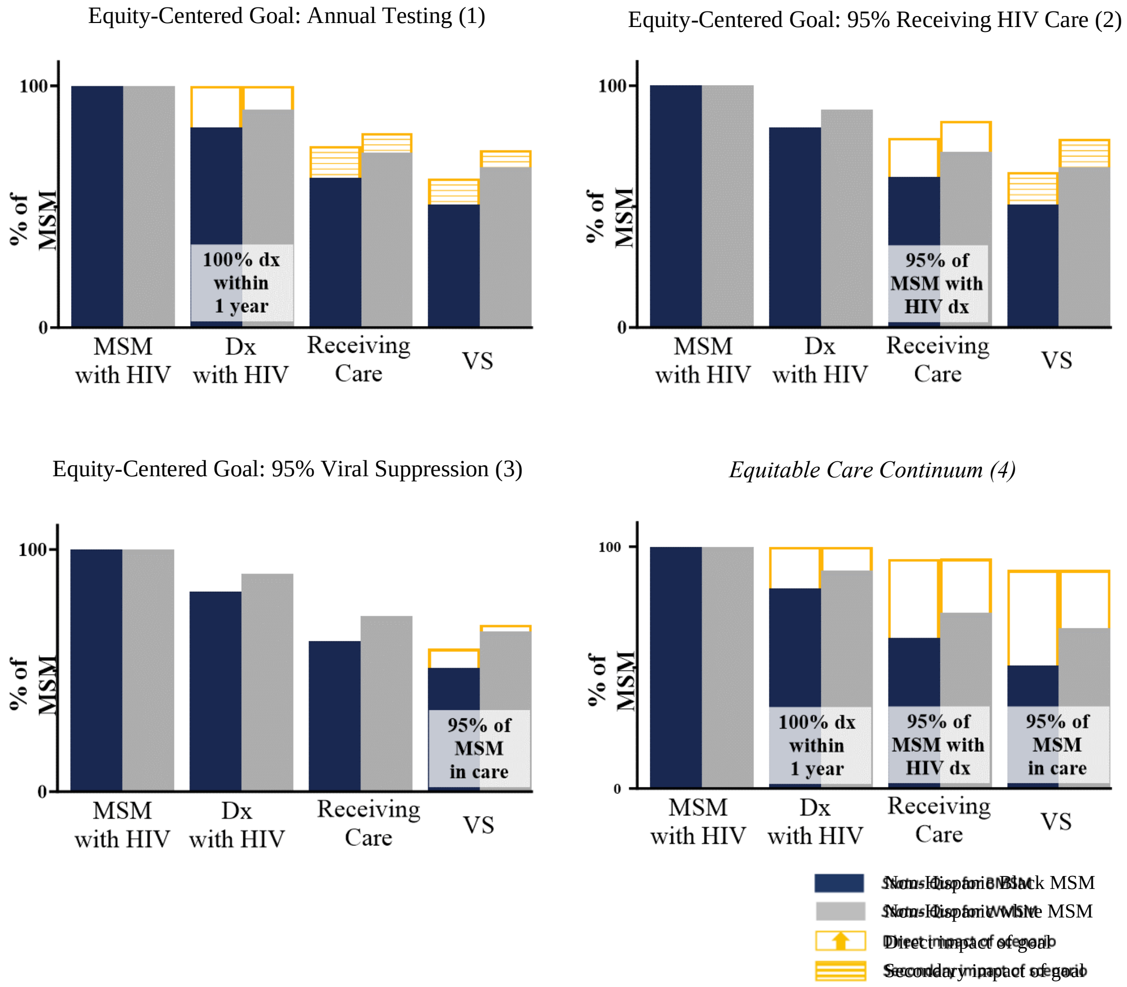

eFigure 2. The HIV Care Continuum Among Non-Hispanic Black and White MSM With HIV in the US With Equity-Centered Goals, and Equitable Care Continuum

© 2023 Rich KM et al. *JAMA Network Open.*

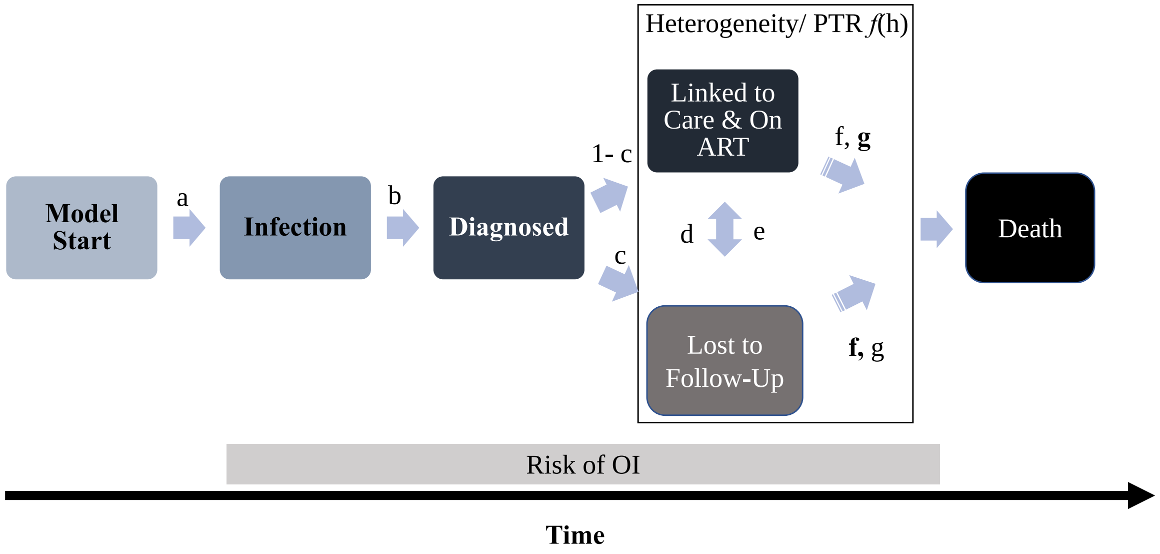

eFigure 3. Model Schematic

© 2023 Rich KM et al. *JAMA Network Open.*
