# Supplementary material for: Projected Life Expectancy Gains From Improvements in HIV Care in Black and White Men Who Have Sex With Men
Source: JAMA Netw Open. 2023 Nov 28;6(11):e2344385. doi: 10.1001/jamanetworkopen.2023.44385 (PMC10685884; doi:10.1001/jamanetworkopen.2023.44385)
Supplement: Supplement 2. — Data Sharing Statement [file jamanetwopen-e2344385-s002.pdf]

Data Sharing Statement

Rich. Projected Life Expectancy Gains From Improvements in HIV Care in Men Who Have Sex With Men. *JAMA Netw Open*. Published November 21, 2023.
doi:10.1001/jamanetworkopen.2023.44385

Data
Data available: Yes
Data types: Other (please specify)
Additional Information: No data were collected for this study. We are happy to make the input parameterization and model based analyses available for review.
How to access data: Please contact Dr. Emily P. Hyle, senior author, at ehyle@mgh.harvard.edu if data sharing is requested.
When available: With publication

Supporting Documents
Document types: Other (please specify)
Additional Information: We are happy to make the input parameterization and model based analyses available for review.
How to access documents: Please contact Dr. Emily P. Hyle, senior author, at ehyle@mgh.harvard.edu if data sharing is requested.
When available: With publication

Additional Information
Who can access the data: Anyone requesting the data.
Types of analyses: For a specified purpose
Mechanisms of data availability: With investigator support and approval of a proposal.
